# The Black Hole of Harlem

**Published:** 1913-10

**Authors:** 


The Black Hole of Harlem.—In June, 1745, one hundred and forty-five English prisoners of war, were by the Swahab of Bengal, India, locked over night in a room, eighteen feet square, with a single small opening. In the morning only eighteen were alive.

That was nearly two centuries ago, in a heathen land. In August, 1913 (thermometer 100 in the shade), twelve negro boys, under twenty years of age, convicts serving short time for minor crimes, were, by two irresponsible guards, locked in a nearly air tight box 7x7x9. They were locked in in the afternoon and next morning they were all dead but four. The cell was dark, of course. There were four small auger holes in the floor, one in each corner, and six overhead, but which did not open into the open air, the box being under a shed. This was on the Harlem plantation, owned by the State of Texas and worked by convicts.

The offense for which these boys were murdered was refusal or inability to pick so many pounds of cotton.

This was in a Christian land, in the twentieth century.

They were smothered, suffocated, and locking up in this box, twelve men, was with the knowledge and consent of the prison physician! On the witness stand during the alleged investigation(?) he said he didn't think it would be prudent to put more than four in such a box!

Let me see. Seven by seven by nine feet is 441 cubic feet of air. Twenty per cent of this is oxygen, that is, 88 cubic feet.

Every person requires and breathes 3000 cubic feet of air (600 cubic feet of oxygen) every hour. Thus, about ten minutes supply for one was the measure for twelve for twenty-four hours. A chicken would have died for want of breath, as these boys did, in less time than that. An investigation (?) followed: No one responsible.

Are we civilized? Can such things be? Are brutal and ignorant guards to be allowed to inflict the death penalty by the cruelest form of torture known, and no one be held responsible for it? Those who authorized this murder are guilty of murder—and it does look as if the responsibility should not be hard to fix, on some "higher-ups"—but—politics; politics doubtless saved them. Politics, like charity, covers a multitude of faults.

These guards or the person just above them, had in their possession permits, signed in blank, to punish at will all who offended. It is intended that these permits, printed forms, shall be issued by the manager only after the physician's certificate that a given convict is able to stand a whipping, for instance; but no convict can stand smothering. The physician, I understand, is still on duty at the seat of the execution.

